# Ketogenic versus Western and standard chow diets favorably alters fat deposition and serum biomarkers in rats

**DOI:** 10.1186/1550-2783-12-S1-P21

**Published:** 2015-09-21

**Authors:** Angelia M Holland, Wesley C Kephart, Ryan P Lowery, Petey W Mumford, C Brooks Mobley, Anna E McCloskey, Joshua J Shake, Paulo Mesquita, Jeffrey S Martin, Andreas N Kavazis, Danielle J McCullough, Michael D Roberts, Jacob M Wilson

**Affiliations:** 1 School of Kinesiology, Auburn University, Auburn, AL, USA; 2 Department of Health Sciences and Human Performance, The University of Tampa, Tampa, FL, USA; 3 Edward Via College of Osteopathic Medicine, Auburn Campus, Auburn, AL, USA

## Background

Very low-carbohydrate (ketogenic) diets are becoming increasingly popular as weight loss interventions. This study examined the effects of ketogenic (KD), Western (WD), and standard chow (StdChow) control diets on fat deposition and serum health-related biomarkers.

## Methods

Male Sprague-Dawley rats (~9-10 weeks of age) were provided isocaloric amounts of either a KD (5.2 kcal/g, 20.2% protein, 10.3% carbohydrate, 69.5% fat; n = 50), WD (4.5 kcal/g, 15.2% protein, 42.7% carbohydrate, 42.0% fat; n = 66), or StdChow (3.1 kcal/g, 24.0% protein, 58.0% carbohydrate, 18.0% fat n = 10) for 6 weeks with daily food intake and body weights recorded. After the animals were sacrificed, 4 different fat depots were weighed and serum was collected in subsets of each diet for further investigation.

## Results

Over the 6-week period, KD rats consumed 3,540 ± 74 (mean±SD) total kcal, WD rats consumed 3,638 ± 83 total kcal, and StdChow rats consumed 3,025 ± 145 total kcal (WD>KD>StdChow; p < 0.001). Remarkably, however, 6-week feed efficiency (g bodyweight gained/kcal consumed) was greater in the WD and (0.042 ± 0.007g/kcal) StdChow (0.045 ± 0.012g/kcal) compared to the KD rats (0.018 ± 0.006g/kcal) (p < 0.001). Total body mass at sacrifice was also significantly less in KD compared to WD and StdChow groups (397 ± 26,494 ± 36 and 472 ± 49g, respectively; p < 0.001). KD and StdChow had significantly less absolute and relative omental (absolute omental: 0.8 ± 0.3g and 1.2 ± 0.4g vs 1.6 ± 0.6g, respectively, p < 0.05; relative omental: 2.1 ± 0.7 and 2.4 ± 0.7 vs 3.2 ± 1.2g/kg, respectively, p < 0.05) compared to WD rats. KD and StdChow also had significantly less perirenal adipose tissue compared to WD (absolute perirenal: 4.2 ± 1.3 and 5.4 ± 1.4 vs 7.8 ± 1.8g, respectively, p < 0.05; relative perirenal: 10.6 ± 2.8 and 11.4 ± 2.4 vs 15.6 ± 3.0g/kg, respectively, p < 0.05). KD had significantly less absolute inguinal subcutaneous (SQ) and scapular brown fat than WD (absolute SQ: 4.3 ± 1.5 vs 6.6 ± 2.4g/kg; absolute brown fat: 0.6 ± 0.2 vs 0.8 ± 0.3g) but similar relative SQ and brown fat weights. Serum triglyceride levels were greater in WD (319.7 ± 109.8mg/dL) versus StdChow rats (163.0 ± 67.0mg/dL; p < 0.05), and both groups presented greater levels versus KD rats (69.9 ± 21.2mg/dL; p < 0.05). Serum cholesterol and glucose levels were significantly less in the KD compared to WD and StdChow rats (cholesterol: 67.7 ± 6.8 vs 89.9 ± 10.8 and 87.0 ± 16.9mg/dL, respectively, p < 0.05; glucose: 166.1 ± 49.6 vs 278.3 ± 99.9 and 256.6 ± 86.9mg/dL, respectively, p < 0.05). White blood cell counts were greater in the WD compared to KD and StdChow groups (15.3 ± 2.6 vs 7.9 ± 2.8 and 9.5 ± 4.3 x10^3 ^cells/µL, p < 0.05), and white blood cell differentials between groups are discussed herein.

## Conclusions

These rodent data suggest that KD is favorable for fat loss and improvements in serum health-related biomarkers compared to WD and even hypocaloric amounts of StdChow.

